# Supplementary material for: Preparation and Performance of Nickel-Doped LaSrCoO3-SrCO3 Composite Materials for Alkaline Oxygen Evolution in Water Splitting
Source: Nanomaterials (Basel). 2025 Jan 28;15(3):210. doi: 10.3390/nano15030210 (PMC11821170; doi:10.3390/nano15030210)
Supplement: Supplementary file 1 [file nanomaterials-15-00210-s001.zip › nanomaterials-3425033-supplementary.pdf]

Preparation and performance of nickel-doped $LaSrCoO_3$-$SrCO_3$ composite materials for alkaline oxygen evolution in water splitting

Bangfeng Zong[a], Xiaojun Pan[b], Lifang Zhang[b], Bo Wei[b], Xiangxiong Feng [b], Miao Guo[b], Duanhao Cao[b], Feng Ye[*,b]

[a]*School of Mechanical and Electronic Engineering, Suzhou University, Suzhou 234000, China*

[b]*Key Laboratory of Power Station Energy Transfer Conversion and System of MOE, School of Energy Power and Mechanical Engineering, North China Electric Power University, Beijing 102206, China*

**Corresponding Author. Tel: +86 10 61773876; fax: +86 10 61773877.*

E-mail address: fye@ncepu.edu.cn (F. Ye)

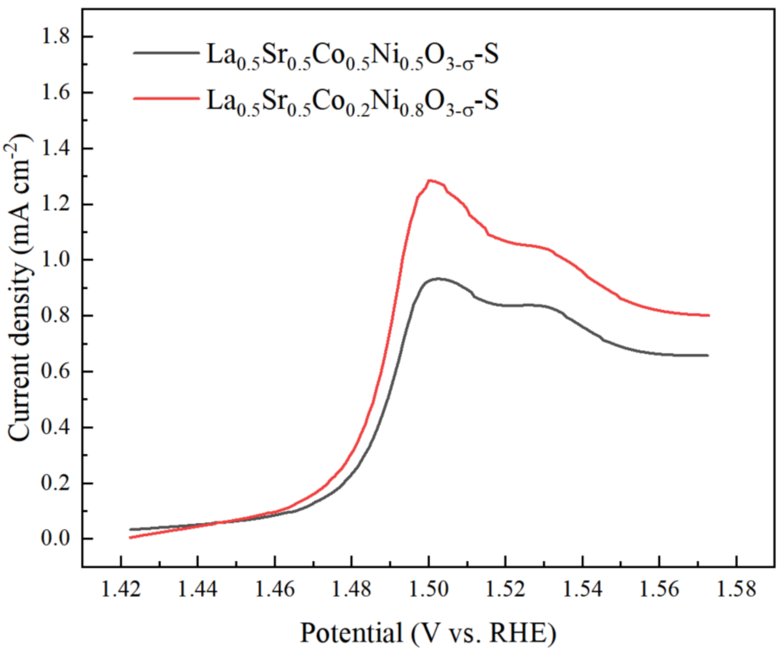

Figure S1. The oxidation peak images of $La_{0.5}Sr_{0.5}Co_{0.5}Ni_{0.5}O_{3-\sigma}$-S and $La_{0.5}Sr_{0.5}Co_{0.5}Ni_{0.8}O_{3-\sigma}$-S

We plotted the oxidation peaks of $La_{0.5}Sr_{0.5}Co_{0.5}Ni_{0.5}O_{3-\sigma}$-S and $La_{0.5}Sr_{0.5}Co_{0.5}Ni_{0.8}O_{3-\sigma}$-S, observing that the $Ni^{2+}/^{3+}$ oxidation peak for $La_{0.5}Sr_{0.5}Co_{0.5}Ni_{0.8}O_{3-\sigma}$-S appears at approximately 1.5 V. However, the peak intensity is very weak, with the current density being only approximately 1 mA cm^{-2}. We believe that the weak intensity of the oxidation peak around 1.5 V is related to the crystal structure and active sites of the catalysts. Nickel-based catalysts with specific crystal structures may hinder the oxidation state transition of nickel, requiring higher potentials for oxidation to occur. Additionally, when the active sites are occupied by other substances, they may hinder the oxidation process of nickel, leading to the disappearance of the oxidation peak. Therefore, we believe that elements such as La, Sr, and Co may occupy more active sites, thereby affecting the appearance of the oxidation peak of Ni.

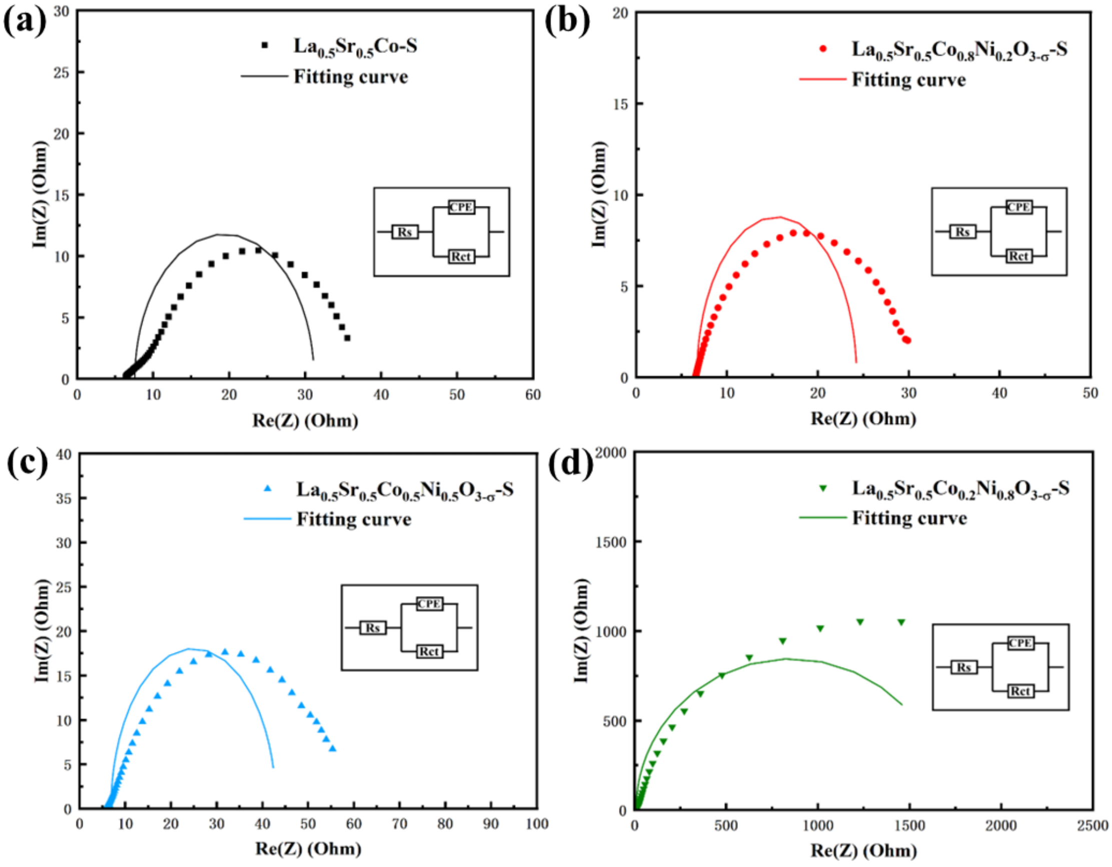

Figure S2. Electrochemical impedance spectroscopy (EIS) plots of $La_{0.5}Sr_{0.5}CoO_{3-\sigma}$-S, $La_{0.5}Sr_{0.5}Co_{0.8}Ni_{0.2}O_{3-\sigma}$-S, $La_{0.5}Sr_{0.5}Co_{0.5}Ni_{0.5}O_{3-\sigma}$-S and $La_{0.5}Sr_{0.5}Co_{0.8}Ni_{0.2}O_{3-\sigma}$-S.

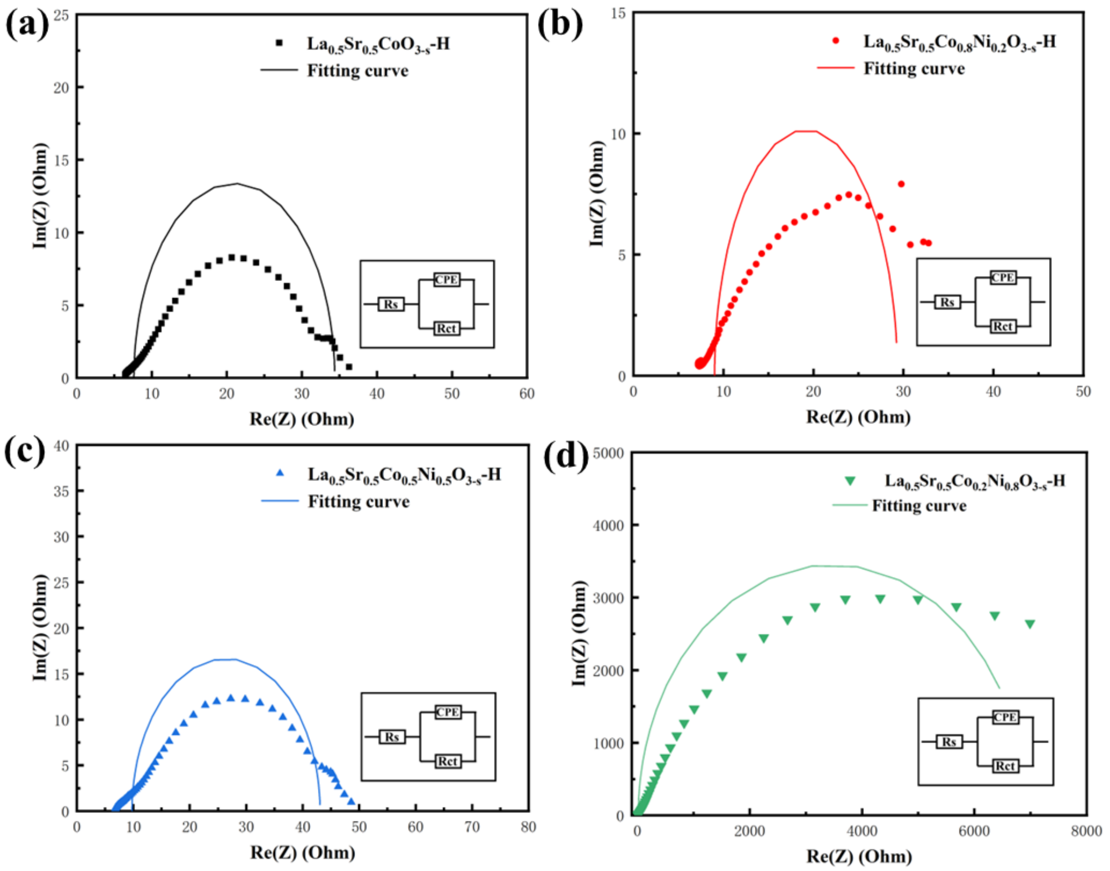

Figure S3. Electrochemical impedance spectroscopy (EIS) plots of $La_{0.5}Sr_{0.5}CoO_{3-\sigma}$-H, $La_{0.5}Sr_{0.5}Co_{0.8}Ni_{0.2}O_{3-\sigma}$-H, $La_{0.5}Sr_{0.5}Co_{0.5}Ni_{0.5}O_{3-\sigma}$-H and $La_{0.5}Sr_{0.5}Co_{0.8}Ni_{0.2}O_{3-\sigma}$-H.

Table S1. Relevant parameters in electrochemical impedance spectroscopy (EIS) fitting.

Samples	Rs (Ohm)	C_2 (mF)	Rct (Ohm)	X^2 (Ohm^2)	X/√N (Ohm)
$La_{0.5}Sr_{0.5}CoO_{3-\sigma}$-S	7.584	1.089	23.58	256.5	2.179
$La_{0.5}Sr_{0.5}Co_{0.8}Ni_{0.2}O_{3-\sigma}$-S	6.676	2.031	17.57	196	1.923
$La_{0.5}Sr_{0.5}Co_{0.5}Ni_{0.5}O_{3-\sigma}$-S	6.892	1.713	36.07	485.9	3.286
$La_{0.5}Sr_{0.5}Co_{0.8}Ni_{0.2}O_{3-\sigma}$-S	7.276	1.892	1690	653426	107
$La_{0.5}Sr_{0.5}CoO_{3-\sigma}$-H	8.762	1.927	33.31	358.2	3.249
$La_{0.5}Sr_{0.5}Co_{0.8}Ni_{0.2}O_{3-\sigma}$-H	9.753	1.009	33.22	545.6	3.016
$La_{0.5}Sr_{0.5}Co_{0.5}Ni_{0.5}O_{3-\sigma}$-H	9.269	1.451	31.29	357.3	3.549
$La_{0.5}Sr_{0.5}Co_{0.2}Ni_{0.8}O_{3-\sigma}$-H	16.13	0.628	6906	7841000	361.5
